# Value of Quantitative and Qualitative Analyses of Circulating Cell-Free DNA as Diagnostic Tools for Hepatocellular Carcinoma: A Meta-Analysis

**DOI:** 10.1097/MD.0000000000000722

**Published:** 2015-04-10

**Authors:** Wenjun Liao, Yilei Mao, Penglei Ge, Huayu Yang, Haifeng Xu, Xin Lu, Xinting Sang, Shouxian Zhong

**Affiliations:** From the Department of Liver Surgery, Peking Union Medical College (PUMC) Hospital, PUMC & Chinese Academy of Medical Sciences, Beijing, 100730, China.

## Abstract

Qualitative and quantitative analyses of circulating cell-free DNA (cfDNA) are potential methods for the detection of hepatocellular carcinoma (HCC). Many studies have evaluated these approaches, but the results have been variable. This meta-analysis is the first to synthesize these published results and evaluate the use of circulating cfDNA values for HCC diagnosis.

All articles that met our inclusion criteria were assessed using QUADAS guidelines after the literature research. We also investigated 3 subgroups in this meta-analysis: qualitative analysis of abnormal concentrations of circulating cfDNA; qualitative analysis of single-gene methylation alterations; and multiple analyses combined with alpha-fetoprotein (AFP). Statistical analyses were performed using the software Stata 12.0. We synthesized these published results and calculated accuracy measures (pooled sensitivity and specificity, positive/negative likelihood ratios [PLRs/NLRs], diagnostic odds ratios [DORs], and corresponding 95% confidence intervals [95% CIs]). Data were pooled using bivariate generalized linear mixed model. Furthermore, summary receiver operating characteristic curves and area under the curve (AUC) were used to summarize overall test performance. Heterogeneity and publication bias were also examined.

A total of 2424 subjects included 1280 HCC patients in 22 studies were recruited in this meta-analysis. Pooled sensitivity and specificity, PLR, NLR, DOR, AUC, and CIs of quantitative analysis were 0.741 (95% CI: 0.610–0.840), 0.851 (95% CI: 0.718–0.927), 4.970 (95% CI: 2.694–9.169), 0.304 (95% CI: 0.205–0.451), 16.347 (95% CI: 8.250–32.388), and 0.86 (95% CI: 0.83–0.89), respectively. For qualitative analysis, the values were 0.538 (95% CI: 0.401–0.669), 0.944 (95% CI: 0.889–0.972), 9.545 (95% CI: 5.298–17.196), 0.490 (95% CI: 0.372–0.646), 19.491 (95% CI: 10.458–36.329), and 0.87 (95% CI: 0.84–0.90), respectively. After combining with AFP assay, the values were 0.818 (95% CI: 0.676–0.906), 0.960 (95% CI: 0.873–0.988), 20.195 (95% CI: 5.973–68.282), 0.190 (95% CI: 0.100–0.359), 106.270 (95% CI: 22.317–506.055), and 0.96 (95% CI: 0.94–0.97), respectively.

The results in this meta-analysis suggest that circulating cfDNA have potential value for HCC diagnosis. However, it would not be recommended for using independently, which is based on the nonrobust results. After combining with AFP, the diagnostic performance will be improved. Further investigation with more data is needed.

## Abstract 2

Supplemental Digital Content is available in the text

## INTRODUCTION

Worldwide, hepatocellular carcinoma (HCC) is the fifth most common cause of cancer and the third largest cause of mortality from cancer-related disease. More than 500,000 new cases of HCC are diagnosed each year.^1^ Although surgical resection is the primary treatment for HCC,^2^ it is not always effective for advanced-stage disease.^3,4^ As with other cancers, early diagnosis of HCC is advantageous for patient outcomes. Although some biomarkers, such as α-fetoprotein (AFP) and Golgi protein, have been used to detect early-stage HCC, the results have not been satisfactory.^5^

Circulating cell-free DNA (cfDNA), a type of cell-free nucleic acids (cfNAs), mostly comes from apoptotic and necrotic cells, or is released from living eukaryotic cells.^6,7^ This naturally occurring biological material can be considered as a new tool for the detection and surveillance of major cancers because abnormal forms of circulating cfDNA are more likely to be present in these patients.^6–8^ These circulating cfDNA abnormalities include quantitative and qualitative changes. Quantitative changes result in higher concentrations of total circulating cfDNA. Qualitative changes include tumor-specific methylation alterations and gene mutations, tumor-specific loss of heterozygosity (LOH), circulating cfDNA strand integrity, circulating nucleosomes, and the presence of viral DNA. All of these alterations have been found in a variety of cancers.^6–8^

The application of circulating cfDNA assay has been reported in a wide range of cancers,^9^ including HCC. Dozens of studies have attempted to determine whether circulating cfDNA is a potential tool for the detection of HCC. However, the results have been varied and have not been systematically evaluated. The purpose of this meta-analysis was to integrate the findings of these published studies and comprehensively evaluate the diagnostic efficiency of circulating cfDNA for HCC.

## METHODS

### Literature Research Strategy and Quality Assessment

The studies included in the meta-analysis were independently retrieved by 2 authors (WJ Liao and PL Ge). The medical subject heading terms (MeSH) and text words included “cell-free DNA,” “circulating DNA,” “plasma DNA,” “serum DNA,” “cfDNA,” “liver neoplasms,” “hepatocellular carcinoma,” “hepatic carcinoma,” “liver tumor,” “liver cancer,” “sensitivity and specificity,” and “accuracy.” They were used to perform a systematic literature search in the PubMed, Web of Science, Cochrane Library, and Embase databases. There was no limit on the start date for published articles, and the search ended in September 2014. For a more comprehensive analysis, we set no language restriction, but the included articles were only in English. When necessary, we also contacted the authors of the articles if more information was needed.

All of the articles that met our inclusion criteria were assessed using QUADAS guidelines. QUADAS is a tool for the quality assessment of diagnostic accuracy studies (maximum score = 14).^10^ In this meta-analysis, the article received a score > 0 if the information that was reported or obtained from the studies was in accordance with QUADAS criteria. If the item did not conform to the criteria or was ambiguous, a score = 0 was recorded.

### Data Extraction

Two reviewers (WJ Liao and HY Yang) independently extracted data from the articles and integrated the final results with input from a third author (HF Xu). The data they extracted from the articles included the lead author's name, publication year, methodological quality score, essential characteristics of the participants, docimastic sample, experimental methods, assay indicators, cutoff values, and sensitivity and specificity data.

### Inclusion Criteria

We included articles that met the following criteria: (a) All evaluation indicators were derived from circulating cfDNA in plasma or serum and (b) sensitivity and specificity values for diagnosis of HCC were reported or could be calculated from the primary data.

### Exclusion Criteria

We excluded articles with any of the following characteristics: evaluation indicators that were derived from circulating plasma or serum cfDNA were not used for HCC diagnosis; (b) the indicators and circulating cfDNA were not related; (c) insufficient data for describing or calculating sensitivity and specificity values; (d) the article included specific evaluation indicators that were studied so rarely that they could not be included in a pooled analysis; and (e) reviews, letters, technical reports, case reports, or comments.

### Subgroups

Three subgroups were evaluated in the meta-analysis:

1. those with abnormal concentrations of total circulating cfDNA (quantitative analysis subgroup);

2. those with tumor-specific single-gene methylation alterations (qualitative analysis subgroup); and

3. those which also included AFP assays for a combined diagnostic indicator (multiple analysis combined with AFP subgroup). In subgroups (1) and (2), circulating cfDNA was the only evaluative indicator for the detection of HCC.

### Statistical Analysis

We used Stata software (version 12.0; Stata Corporation, College Station, TX) to perform the meta-analysis. Pooled sensitivity and specificity, positive likelihood ratio (PLR), negative likelihood ratio (NLR), diagnostic odds ratio (DOR), corresponding 95% confidence intervals (CIs), and the confidence and prediction contours of the summary receiver operating characteristic (SROC) curves were calculated using a bivariate generalized linear mixed model, to analyze the test accuracy.^11–14^ The area under the curve (AUC) was used for grading the overall accuracy as a potential summary of the SROC curve.^15,16^ The χ^2^ test and Wilcoxon rank sum test was used to compare the pooled sensitivity and specificity, PLR and NLR between different subgroups in this meta-analysis, respectively.

Heterogeneity among these studies was verified using the of likelihood ratio test (LRT)_I^2^ statistic^17^ and LRT_Q (χ^2^) statistics. I^2^ ≥ 50% or *P* < 0.10 for LRT_Q statistics indicates substantial heterogeneity. Meta-regression analysis can be used to explore the sources of heterogeneity.^18^ The 7 variables in this meta-analysis were “publication year,” “study location,” “number of subjects,” “docimastic sample,” “experimental methods,” “cutoff values,” and “abnormal methylated genetic locus.”

We performed the Egger test and generated funnel plots to examine potential publication bias.^19^ A nonparametric “trim and fill” analysis^20^ was also performed because the number of studies was not sufficient for an independent analysis of publication bias. For each analysis, a result was considered to be statistically significant if the *P* value was <0.05.

## RESULTS

### Characteristics of the Studies

**FIGURE 1:**
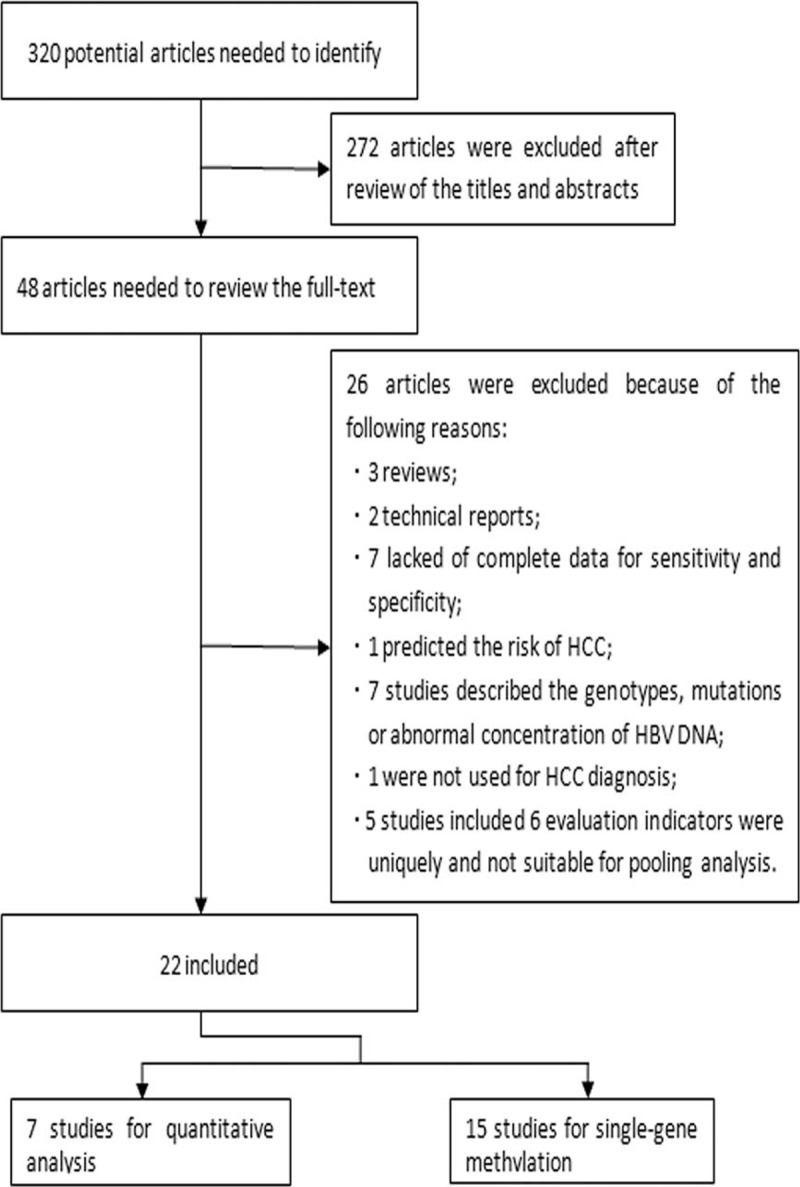
Flowchart for inclusion and exclusion of studies in the meta-analysis. HCC =  hepatocellular carcinoma.

A total of 22 studies^21–42^ were included in the meta-analysis, with 2424 subjects in total, including 1280 patients with HCC. The 1144 patients without HCC were included in these studies as control groups, which comprised healthy volunteers, or chronic hepatitis (HBV-related or HCV-related) or cirrhosis patients. Most of these studies had presented a composite control population. The flowchart for the inclusion and exclusion of these studies is presented in Figure 1. Most of the subjects were from Asia, with the remaining patients from Italy,^21^ the United States,^33^ and Egypt.^36^ All the trials were prospective studies.

Of the 22 studies, 7^21–27^ evaluated abnormal concentrations of circulating cfDNA in plasma or serum (quantitative analysis group). Fifteen trials^28–42^ assessed the validity of using single-gene methylation alterations (qualitative analysis group). Six articles indicated that sensitivity and specificity tended to increase after AFP was included as a diagnostic indicator.^25,27,28,33,34,40^ The detailed results are presented in Table 1. In addition, the use of AFP assay as a control subgroup for HCC diagnoses only was assessed in 8 of these studies.^21,22,27,28,32–34,42^ (Supplementary Table 1, http://links.lww.com/MD/A243).

**TABLE 1:**
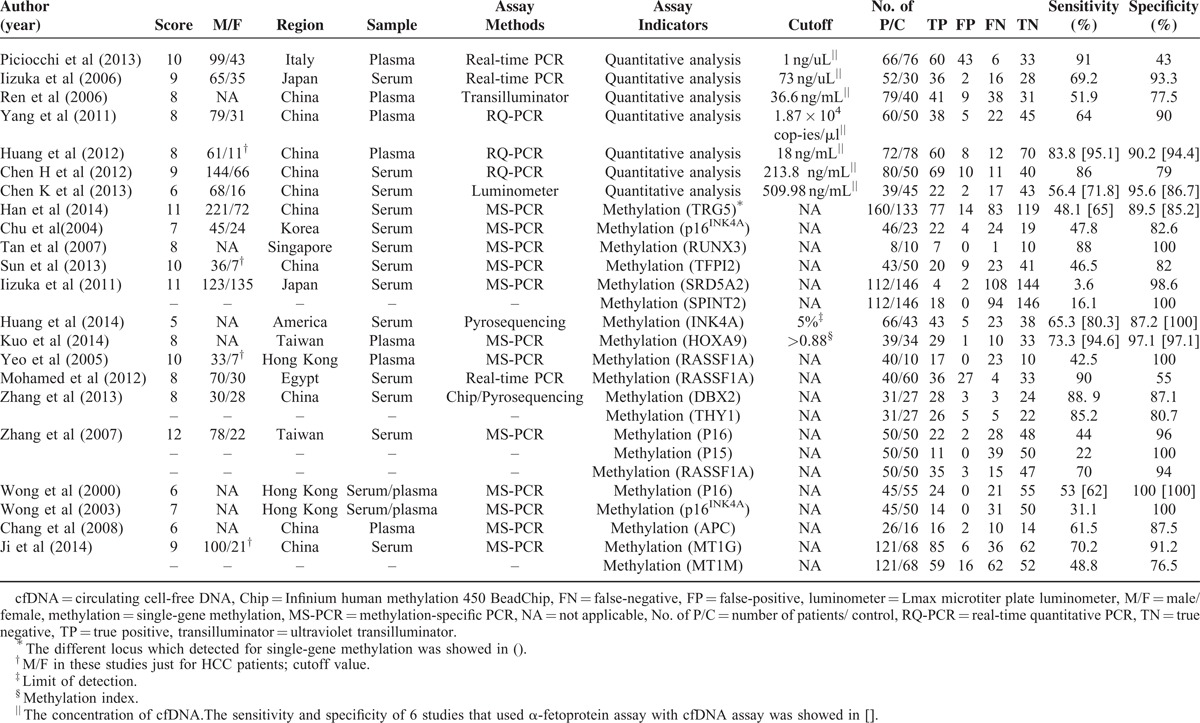
Summary of Studies Included in the Meta-Analysis

Some articles required additional explanation. Kuo et al had described 40 HCC patients in their study, 1 of whom was excluded because of an unclear final diagnosis.^34^ Therefore, the data in the experimental group were adjusted from 40 to 39. Chang et al chose APC hypermethylation as an indicator only because the result was statistically significant.^41^ Other evaluation indicators that were included were allelic imbalances,^23^ tumor-associated copy number aberrations,^43^ serum DNA integrity,^44^ frequent LOH of microsatellite markers in serum,^45^ genome-wide hypomethylation,^43^ multi-gene methylation,^46^ and circulating mitochondrial DNA.^47^ Because these individual indicators were not used in sufficient numbers of studies, pooled analysis could not be performed. The same was true for the diagnostic indicators combined with prothrombin-induced vitamin K absence II (PIVKA-II) and a-L-fucosidase, which were only described in the studies of Iizuka et al^32^ and Chen et al,^27^ respectively. Furthermore, in order to reflect the ideal control population more objectively, all control subjects described in these studies would be included in this meta-analysis.

### Diagnostic Accuracy

Pooled sensitivity and specificity, PLR, NLR, and DOR are used to estimate the diagnostic accuracy in the meta-analysis of the diagnostic test. PLR indicates the amount by which the odds of disease would increase for a positive test and NLR indicates the amount by which the odds of disease would decrease for a negative test. That is to say, a greater numerical value of PLR indicates a higher probability of a true-positive (TP) when the test is positive. Analogously, the probability of a true-negative and the numerical value of NLR are an inverse ratio when the test is negative. The DOR value is an estimate of the effect and is the ratio of the odds of positivity in the diseased relative to the odds of positivity in the nondiseased.^38^ A higher numerical value for DOR indicates better discriminatory test performance. Hence, we used these indicators to estimate the diagnostic accuracy among these subgroups.

Pooled sensitivity and specificity for the quantitative analysis were 0.741 (95% CI: 0.610–0.840) and 0.851 (95% CI: 0.718–0.927), respectively. The calculated value for PLR was 4.970 (95% CI: 2.694–9.169), and NLR was 0.304 (95% CI: 0.205–0.451). The DOR value was 16.347 (95% CI: 8.250–32.388).

Overall results for the subgroup of qualitative analysis were also estimated. The value for pooled sensitivity was 0.538 (95% CI: 0.401–0.669), specificity was 0.944 (95% CI: 0.889–0.972), PLR was 9.545 (95% CI: 5.298–17.196), NLR was 0.490 (95% CI: 0.372–0.646), and DOR was 19.491 (95% CI: 10.458–36.329).

All of these results in these 2 subgroups indicated that a high level of accuracy was presented. But regrettably, a high probability of true-negative error rate would influence the robustness of diagnosis.

**FIGURE 2:**
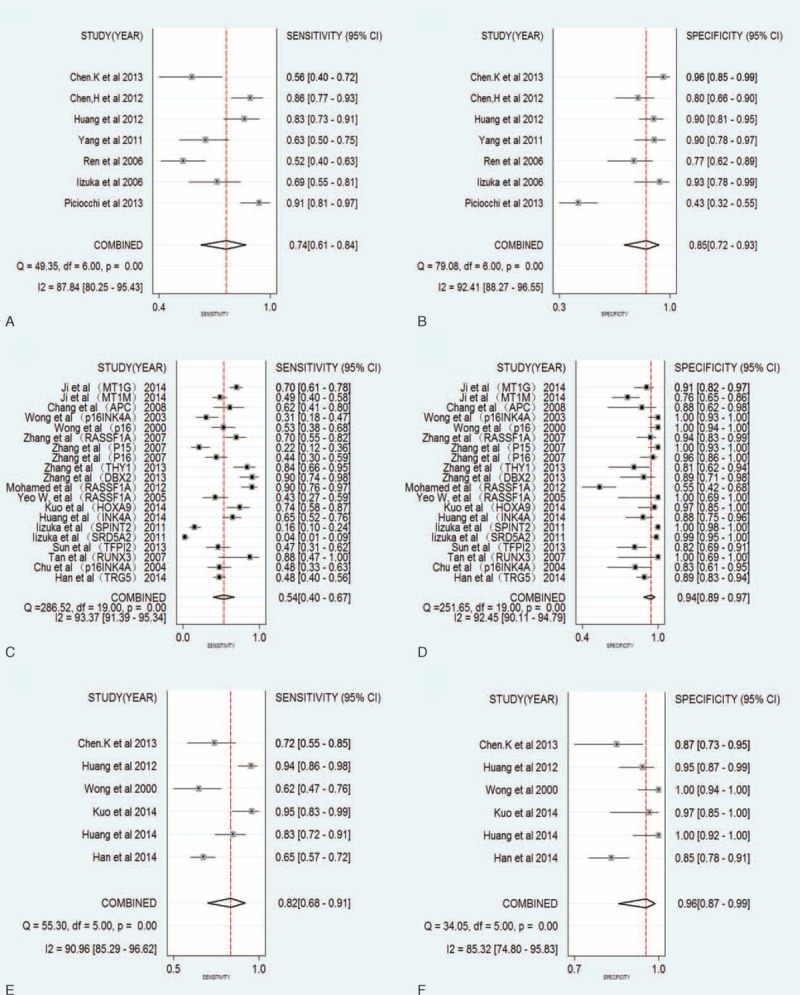
Forest plots of estimates of sensitivity and specificity for the different subgroups. The point estimates (brown squares) and 95% CIs (error bars) for studies in different subgroups are presented in these Forest plots. (A and B) Forest plots for the quantitative analysis subgroup with the diagnostic indicator of abnormal concentration of total circulating cfDNA; (C and D) Forest plots for the qualitative analysis subgroup. This group of studies used tumor-specific single-gene methylation alterations as the evaluation indicator; (E and F) Forest plots for the multiple analysis subgroup (ie, combined with [AFP] assay). The sensitivity and specificity values in this group were re-estimated after adding AFP. AFP = α-fetoprotein, cfDNA = circulating cell-free DNA, CI = confidence interval.

However, when the AFP assay was added as a combined diagnostic indicator, all results improved—the values for pooled sensitivity and specificity were 0.818 (95% CI: 0.676–0.906) and 0.960 (95% CI: 0.873–0.988), respectively. PLR was 20.195 (95% CI: 5.973–68.282), NLR was 0.190 (95% CI: 0.100–0.359), and DOR was 106.270 (95% CI: 22.317–506.055). The results for the Forest plots are presented in Figure 2.

In addition, we also estimated the diagnostic accuracy of AFP assays in this meta-analysis. Pooled sensitivity and specificity were 0.523 (95% CI: 0.415–0.628) and 0.909 (95% CI: 0.794–0.963), PLR was 5.768 (95% CI: 2.576–12.915), NLR was 0.525 (95% CI: 0.427–0.645), and DOR was 10.990 (95% CI: 4.598–26.267), respectively. (Supplementary Figure 1, http://links.lww.com/MD/A243).

**FIGURE 3:**
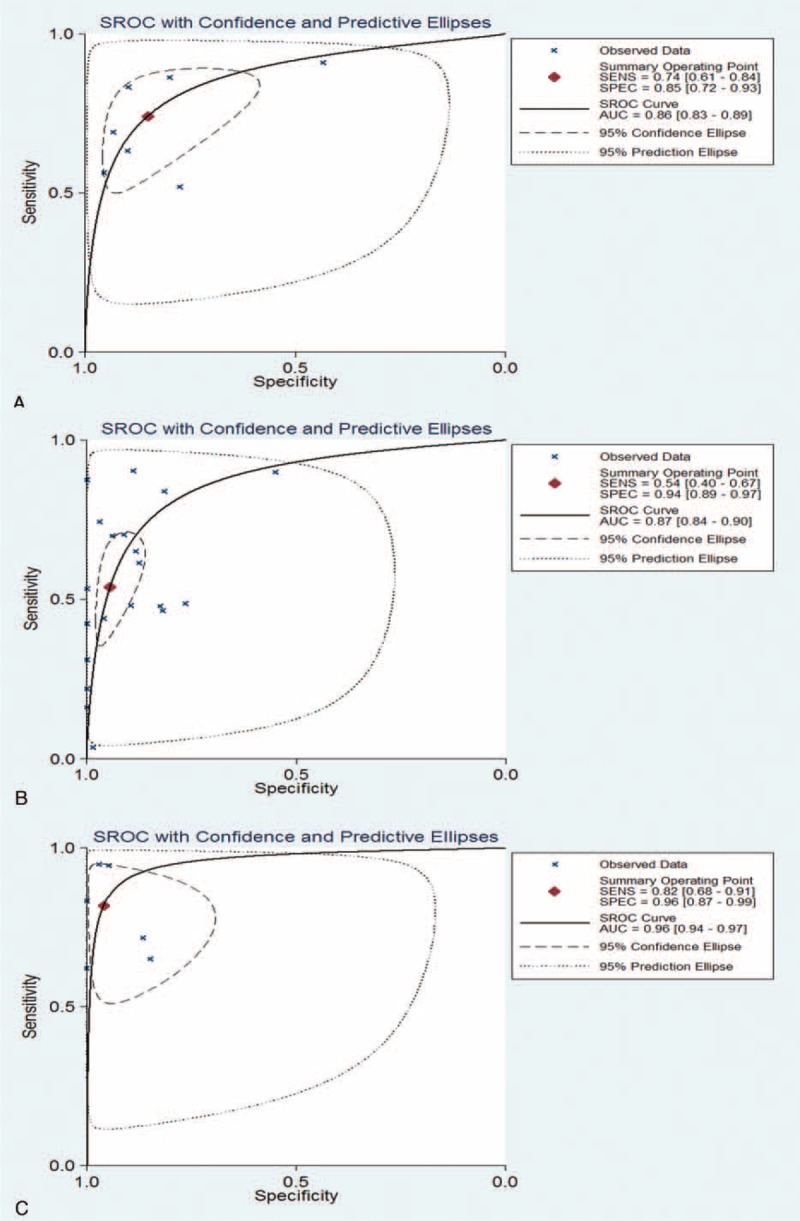
SROC curves for qualitative, quantitative analysis and multiple analysis (combined with AFP assay). (A) SROC curves for the subgroup of quantitative analysis; (B) SROC curves for the subgroup of qualitative analysis; (C) SROC curves for multiple analysis (combined with α-fetoprotein (AFP) assay). The confidence ellipse indicates that the mean values for sensitivity and specificity were more likely to be in this region. The prediction ellipse (increased uncertainty) indicates that individual values for sensitivity and specificity were more likely to be in this region. AFP = α-fetoprotein, AUC = area under the curve, SROC = summary receiver operating characteristic.

Estimation of the SCOR curve is 1 method that can be used in meta-analysis to estimate overall diagnostic performance. It can demonstrate the trade-off between sensitivity and specificity values in multiple studies.^14^ In Figure 3, the observed data, together with the confidence and predictive ellipses, are presented in graphs of the SROC curves. In the quantitative analysis subgroup (Figure 3A), the AUC was 0.86 (95%CI: 0.83–0.89), which indicated a higher level of moderate overall accuracy.^15^ The LRT_I^2^ statistic value was 96.9 (95% CI: 94.70–99.09), indicating that an evident heterogeneity was present in these 7 studies. The LRT_Q (χ^2^) statistic was 64.42 (*P* = 0.000), indicating that the heterogeneity was likely the result of nonthreshold effects.

The SROC curve for the qualitative analysis are presented in Figure 3B. The AUC was 0.87 (95% CI: 0.84–0.90), the LRT_I^2^ statistic was 99.23 (95% CI: 98.89–99.58), and the LRT_Q (χ^2^) statistic was 261.227 (*P* = 0.000). These results indicated a slightly improved diagnostic accuracy, compared with quantitative analysis. However, an increased heterogeneity derived from nonthreshold effects was also revealed by the analysis.

The results for the subgroup of multiple analysis combined with AFP assay indicated the best overall diagnostic performance (Figure 3C). The AUC was 0.96 (95% CI: 0.94–0.97), the LRT_I^2^ statistic was 57.43 (95% CI: 3.98–100), and the LRT_Q (χ^2^) statistic was 4.698 (*P* = 0.048).

### Heterogeneity and Meta-Regression Analysis

**FIGURE 4:**
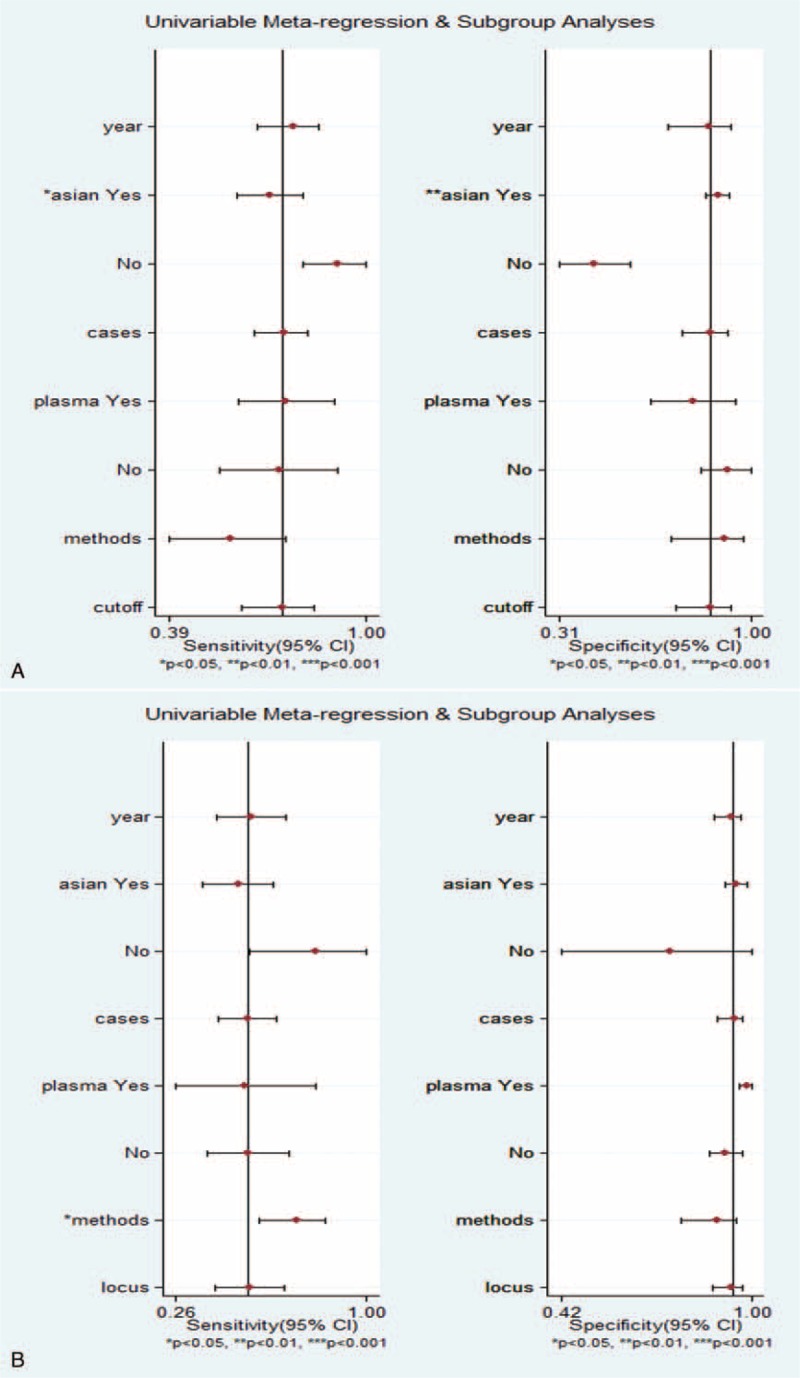
Forest plots of meta-regression analyses for sensitivity and specificity in the subgroup of (A) quantitative analysis and (B) qualitative analysis. CI = confidence interval.

Regardless of the indicator used, obvious heterogeneity from nonthreshold effects was present in these studies. To find the source of the heterogeneity, we used meta-regression analysis to assess covariates used in the studies. The study characteristics included “publication year (Year),” “study location (Region: Asia or not),” “number of subjects (Cases),” “docimastic sample (Sample: Plasma or Serum),” “experimental methods (methods),” “cutoff value (Cut-off),” and “genetic locus (Locus)” (Table 1). The meta-regression analyses are presented in Figure 4. The results suggested that the “study location” covariate might produce major heterogeneity in the subgroup of quantitative analysis, and the covariate of “assay methods” might be potential sources of heterogeneity in sensitivity in the qualitative analysis subgroup.

### Publication Bias

**FIGURE 5:**
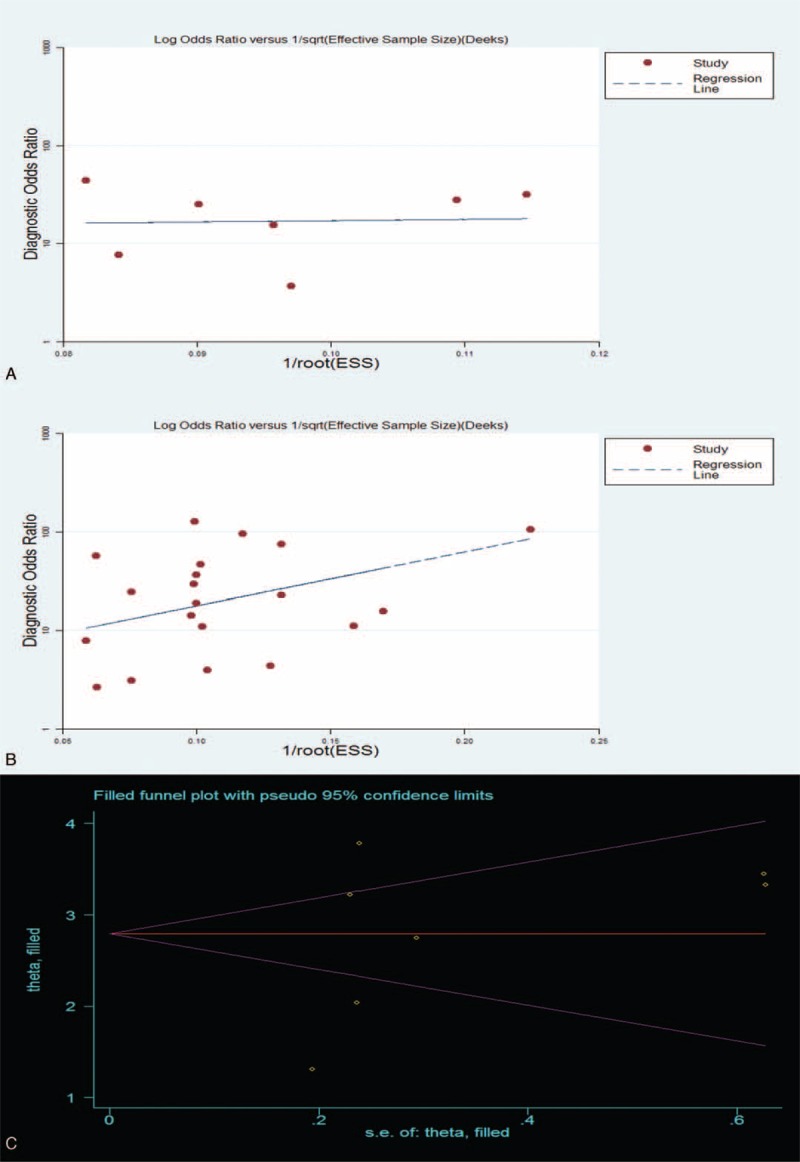
Funnel plots for the assessment of potential publication bias in the qualitative and quantitative analysis subgroups. (A) Funnel plots for the subgroup of quantitative analysis. (B) Funnel plots for the subgroup of qualitative analysis. The funnel graph indicates the results for the linear regression of the log odds ratios on the inverse root of the effective sample sizes. Each solid circle represents a study included in the meta-analysis. The line in the center indicates the summary DOR. (C) Funnel plots for the assessment of potential publication bias in the qualitative analysis subgroup using nonparametric “trim and fill” analysis. The funnel graph is a plot of the log of the DOR against the SE of the log of the DOR. “Theta” is the effect estimate and is the log DOR in this graph. DOR = diagnostic odds ratio, ESS = effective sample sizes.

The Egger test, a linear regression of log odds ratios on the inverse root of the effective sample sizes, is used to test the funnel plot asymmetry in diagnostic meta-analyses.^19^ The funnel plot for the quantitative subgroup (Figure 5A) had a coefficient of 3.04 (95% CI: −84.96–91.05) and the *P*-value of 0.933, which indicated that the funnel plot was symmetric. Publication bias was not present in these studies.

However, the Egger test was not particularly suitable for such a small number of studies (7). Therefore, we performed the extra nonparametric “trim and fill” analysis to verify the conclusion of publication bias. This method, which was proposed by Duval and Tweedie, can be used to estimate the number of missing studies and the outcome after adjusting for and incorporating theoretical missing studies.^19^ The pooled OR point estimates were 2.536 (95% CI: 2.338–2.735) for the fixed effect model and 2.794 (95% CI: 2.006–3.582) for the random effects model before trimming the missing studies (*P* < 0.001). After the “trim and fill” process was completed, none of the studies were trimmed and the final data were unchanged. The results indicated that no publication bias was present in these studies. The results for the symmetrical funnel plot are presented in Figure 5C. However, the outcome after adjusting and filling was very different. The pooled OR point estimates changed to 12.632 (95% CI: 10.356–15.408) for the fixed-effect model and 16.347 (95% CI: 7.434–35.949) for the random-effects model (*P* = 0.000). These results indicated that the conclusion about publication bias in the 7 studies was not robust.

The Egger test results for qualitative analysis are presented in Figure 5B. The coefficient was 12.6 (95% CI: −6.86–31.99), and the *P*-value was 0.191. These results indicated that the funnel plot was symmetric and that publication bias was not present.

## DISCUSSION

As patients with HCC would clearly benefit from its diagnosis at the early stage, biomarkers for HCC have been widely studied, but few have satisfactory performance for clinical use. However, circulating cfDNA has attracted more attention than other biomarkers and has led to dozens of studies. Hence, the goal of this meta-analysis was to integrate these published results for the first time and systematically evaluate the diagnostic performance of circulating cfDNA.

The results of the meta-analysis indicated that addition of the AFP assay as a combined diagnostic indicator presented the optimal pooled sensitivity and specificity. The numerical values were 0.818 (sensitivity) and 0.960 (specificity). As control subgroup, the sensitivity value of AFP assay was 0.523, which was similar to the result of Farinati et al (the sensitivity was 0.54).^48^ HCC diagnostic sensitivity in the quantitative analysis subgroup (χ^2^ = 50.3, *P* < 0.001) and combined-AFP analysis subgroup (χ^2^ = 88.74, *P* < 0.001) was superior to AFP assay alone, although not for the subgroup of qualitative analysis (χ^2^ = 0.343, *P* = 0.558). We further performed the comparison between the 2 subgroups of quantitative and qualitative analysis, and found that the former had a better sensitivity (χ^2^ = 53.71, *P* < 0.001). The reason why the ability of diagnosis for qualitative analysis was not so excellent might be that some genetic loci that were chosen for detection had an advantage in non-HCC patients.^32,38^

Regardless of the type of diagnostic indicator chosen, the pooled specificity values were acceptable. This pooled specificity in qualitative analysis subgroup was high and did not significantly vary from the multiple analysis subgroup (χ^2^ = 1.33, *P* = 0.248). These respective values were superior to those of the quantitative analysis subgroup (χ^2^ = 31.62, *P* < 0.001 and χ^2^ = 25.27, *P* < 0.001) and AFP assay-only group (χ^2^ = 7.11, *P* = 0.008 and χ^2^ = 8.534, *P* = 0.003). To further evaluate diagnostic accuracy, we analyzed DOR, PLR, NLR, AUC, and the SROC curves.

The DOR reflects test performance and the values range from zero to infinity. The capacity of discrimination and the numerical value of DOR are a direct ratio. In our results, all DOR values indicated that a high level of accuracy was present. The subgroup that included AFP had the best discriminatory test performance (DOR was 106.270).

The SROC curve and the corresponding AUC may be used in meta-analysis to estimate the overall diagnostic performance. The following evaluation criteria have been suggested: low (AUC: 0.5–0.7), moderate (AUC: 0.7–0.9) or high (AUC: 0.9–1.0) accuracy.^15^ In this meta-analysis, the AUC values for the qualitative and quantitative analyses were 0.86 and 0.87, respectively. These values indicated a higher part of the moderate overall accuracy category. The AUC value for the subgroup that included AFP was 0.96, indicating the best overall diagnostic performance.

Although these results showed a high level of accuracy, their unsatisfactory likelihood ratios indicated poor robustness. LRs are metrics that can reflect the verity of sensitivity and specificity. In our meta-analysis, the PLR was 4.970 and NLR was 0.304 in quantitative analysis. This result indicated that an approximately four to five times greater chance of a TP would be indicated by a positive test result, and an approximately 30% error rate would be present when the TN was determined in the negative test. The PLR was 9.545 and the NLR was 0.490 for the qualitative analysis subgroup. It might produce a higher true-negative error rate because the numerical value of NLR was so poor, although a higher pooled specificity had been presented. In other words, a negative cfDNA assay result should be interpreted with caution when single-gene methylation is used independently for the detection of HCC. These numerical values had no statistical significance in these 2 subgroups (*z* = −0.969, *P* = 0.333 and *z* = −1.384, *P* = 0.166). However, after adding AFP as a combined diagnostic indicator, the results for PLR and NLR clearly improved to 20.195 and 0.190, respectively. Addition of AFP improved the discrimination capability for HCC diagnosis and the result was more robust when the test was negative.

We were also concerned about the effect of publication bias. The results could have been biased if positive results were more likely to be published. However, the funnel plot and the Egger test results did not indicate a publication bias. We also used nonparametric “trim and fill” analysis for the quantitative subgroup analysis. The results did not change. Furthermore, we performed meta-regression analysis and found that the covariates of “study location” and “assay methods” might be potential sources of heterogeneity in our meta-analysis.

Our meta-analysis had some limitations. First, it was impossible for us to determine all sources of heterogeneity. We did not include some covariates because the required data were not available from the selected articles. These probable covariates included tumor size, metastasis, and TNM staging. Second, although we performed a thorough literature search, a smaller number of studies (ie,7) were included in the quantitative analysis subgroup. Consequently, some results of the pooled analysis were not robust. More studies are needed for future analyses. The final limitation was that the inclusion of only English-language studies might have introduced some bias to the analysis.

The consistent results for the circulating cfDNA assay increased confidence that it may be useful for the detection of cancer. The hypothesis of this meta-analysis was that that circulating cfDNA assay is valuable for the detection of HCC, and the results indicated that the diagnostic accuracy was great. However, the unsatisfactory LRs indicated that this biomarker was not robust enough and we do not recommend its use as a stand-alone diagnostic tool. We also found that the diagnostic performance of the circulating cfDNA assay can be improved by using it in combination with the AFP assay. This encouraging result might support the application of circulating cfDNA for the diagnosis of HCC in future clinical practice. Otherwise, we also believed that novel methodologies with new technologies would open up the possibility of screening for methylation variation and genotype quantification in cfDNA as a tool for the detection of different types of cancers. More studies are needed to confirm the results of our meta-analysis.

## CONCLUSION

The results of this meta-analysis suggested that assay of circulating cfDNA may be used in HCC detection because the assay has a higher level of moderate diagnostic accuracy. However, the results of the cfDNA assay analysis lacked robustness, so we recommend that the assay should not be used for independent application. Extra caution should be exercised if only the single-gene methylation detection assay is used for HCC diagnosis. The diagnostic accuracy, robustness, and heterogeneity improve when AFP assay is used in combination with the other assays. Use of circulating cfDNA assay combined with AFP assay may improve the diagnostic performance for the detection of HCC.
